# Effect of non-synonymous SNP on JAK1 protein structure and subsequent function

**DOI:** 10.6026/97320630015723

**Published:** 2019-10-26

**Authors:** Rozario Liza, Sharker Tanima, Nairuz Tahsin, Morshed Md Reaz

**Affiliations:** 1 Department of Biochemistry and Molecular Biology, Noakhali Science and Technology University, Bangaldesh-3814

**Keywords:** JAK 1 gene, non synonymous SNP, structure prediction, altered function

## Abstract

JAK1 gene plays a critical role in signalling. Malfunction of JAK1 is linked to numerous human diseases ranging from chronic inflammation to cancers and autoimmune diseases. Genetic variations in JAK1 exhibit deleterious effects on gene function leading to the deregulation of signalling pathways. A comprehensive list of nsSNPs potentially affecting the structure and function of JAK1 gene is not available. We report 3 deleterious nsSNPs (F78L, Q644R and S646F) in the coding region of JAK1 with predicted structure and function linking to diseases. However, further studies are needed to validate this preliminary observation.

## Background

The Janus kinases (JAKs) are one of ten recognized families of non-receptor protein tyrosine kinases that constitute a novel type of signal transduction pathway activated in response to cytokines and interferons. They govern many essential cellular functions such as survival, growth, development, apoptosis and immune regulation [1]. Activation of JAK in response to ligand involves receptor dimerization and phosphorylation creating docking sites for signal transducers and activators of transcription (STATs). Thus, activation of STAT proteins plays an important role in JAK kinase function. Mammals have four members of this family: JAK1, JAK2, JAK3 and Tyrosine Kinase 2 (TYK2). Each member possesses a C-terminal protein tyrosine kinase domain, an adjacent kinase-related domain and five further domains extending towards the N terminus which have amino acid similarity between members of this family [2]. In humans, the JAK1 gene is located on chromosome 1p31.3. JAK1 plays a significant role in lymphoid cell precursor proliferation, survival and differentiation. JAK1 somatic mutations occur in individuals with acute lymphoblastic leukemia (ALL) [3]. JAK1 loss of function results in a deficit in the production of mature B lymphocytes due to a block in differentiation at the pro–B/pre–B cell transition step [4]. JAK1 gene mutations are hypothesised as initial molecular defects in human cancer and autoimmune diseases [5]. Two different heterozygous mutations in the JAK1 gene can contribute to complete loss of the protein in several different prostate cancer cell lines [6].

Understanding the human genetic variation is one of the major challenges to analyse the differences in susceptibility to diseases and designing individualized therapeutic treatments. It was estimated that 90% of human genetic variations were caused by single nucleotide polymorphisms (SNPs). SNPs are often neutral while some of them contribute to disease predisposition by modifying protein function. The non-synonymous single nucleotide polymorphisms (nsSNPs) trigger genomic disparities in the gene coding regions forming about half of all genetic changes related to human inherited diseases. The nsSNPs are responsible for amino acid substitutions resulting in functional variations of proteins in humans. Functional diversity may have deleterious or neutral effects on protein structure or function. Damaging effects might include destabilization of protein structure and dynamics, altering gene regulation, affecting protein charge, geometry, hydrophobicity, translation and protein-protein interactions [7].

Numerous efforts have been implemented to illustrate the deleterious effects of nsSNPs on the stability of proteins linking to its function. Computer aided prediction tools have been widely applied to identify the effect of deleterious SNPs in candidate genes based on the biochemical severity of the amino acid substitution towards phenotype implications [8]. The functional consequences of most of the nsSNPs in JAK1 are still unknown at the structural level. Therefore, it is of interest to investigate the nsSNPs in the JAK1 gene and the effect on the protein structure and consequent function using prediction models. The deleterious nsSNPs were further analysed for protein stability linking to altered molecular function using computer aided known data enabled prediction tools.

## Methodology

### Retrieval of SNPs:

Data on SNPs in human JAK1 gene was collected from the dbSNP-NCBI (http://www.ncbi.nlm.nih.gov/SNP/). The FASTA format of the protein sequence of JAK1 gene was obtained from the UniProtKB database (http://www.uniprot.org/uniprot/).

### Functional consequence of nsSNPs distinguishing intolerant from tolerant (SIFT):

SIFT (https://sift.bii.a-star.edu.sg/) is a predicting tool for analysing the effects of an amino acid substitution on protein function. SIFT is used to the human variant databases to distinguish mutations (deleterious) involved in disease from neutral polymorphisms.The rsIDs of each nsSNP of JAK1 gene were submitted as a query [9].

### Functional impacts of nsSNPs by screening for non-acceptable polymorphisms (SNAP2):

SNAP2 (https://www.rostlab.org/services/SNAP/) is a program to predict the functional effects of nsSNPs by differentiating between effective and neutral variants. The FASTA format of protein sequences is used as an input for SNAP2 [10].

### Functional effects of nsSNPs using PolyPhen version 2:

PolyPhen-2 (Polymorphism phenotyping) (http://genetics.bwh. harvard.edu/pph2/) scores the impact of amino acid substitutions on the stability and function of proteins. The prediction output is classified as probably damaging, possibly damaging, and benign with specificity and sensitivity values for a mutation [11].

### Functional impacts of nsSNPs using PROVEAN:

PROVEAN (Protein variation effect analyzer) (http://provean.jcvi.org/index.php) predicts the functional impact of protein sequence variations as 'deleterious' or 'neutral' by assessing the single amino acid substitutions.FASTA format data with substitutions predicted by the SIFT server is used as an input [12].

### Disease related nsSNPs using SNPs and GO, PhD-SNP and PANTHER:

SNPs and GO (http://snps.biofold.org/snps-and-go/snps-and-go.html), PhD-SNP (Predictor of Human Deleterious Single Nucleotide Polymorphisms) and PANTHER (Protein Analysis through Evolutionary Relationships) assigns nsSNP to diseases. The FASTA format of the protein sequence is used as an input [13].This server also provides the output result for additional two servers such as PhD-SNP [14] and PANTHER [15] algorithms.

### Protein stability changes on nsSNPs using I-Mutant 3.0:

I-Mutant 3.0 (http://gpcr2.biocomp.unibo.it/cgi/predictors/I-Mutant3.0/I-Mutant3.0.cgi) predicts protein stability changes by single point mutation. It peculates the value of the free energy stability change by nsSNPs using protein structure or sequence data [16].

### nsSNPs impact on surface and solvent accessibility of protein using NetSurfP:

The FASTA sequence of JAK1 protein was submitted to NetSurfP (http://www.cbs.dtu.dk/services/NetSurfP/) to predict secondary structure, surface, and solvent accessibility of amino acids. The outcome shows buried (low accessibility), partially buried (moderate accessibility) and exposed (high accessibility) region in protein structure [17].

### Structure to function assignment of nsSNPs using project HOPE:

HOPE (Have (y) Our Protein Explained) (https://www3.cmbi.umcn.nl/hope) hypothesizes the effects of the mutation on the 3D structure and the corresponding function. HOPE collects information from data on the 3D coordinates of the protein from PDB, sequence annotations from the UniProt database and DAS prediction [18].

## Results and Discussion:

A dataset of SNPs in human JAK1 gene (gene ID: 3716) was retrieved from the dbSNP database. A total of 52315 SNPs were found in human JAK1 gene and 1957 of them were reported in the exon region of the gene. Among them 483 (473 missense and 10 nonsense) were non-synonymous SNPs, which contribute to only 0.92% of all SNPs known in human JAK1 gene. Different computational algorithms such as SIFT,SNAP2, Polyphen-2,PROVEAN,SNPs and GO, PhD-SNP and PANTHER were used for finding the deleterious nsSNPs. SIFT was used for the preliminary screening and identification of functionally significant nsSNPs.SIFT classifies missense variant as tolerated (equal to 0.05) or deleterious (less than 0.05). SIFT identified 30 substitutions as tolerated and 20 as deleterious from 483 nsSNPs (Table 1).It should be noted that the rest of the rsIDs were not found by SIFT [9].SNAP2 and PolyPhen-2 were used to support the data obtained from SIFT. SNAP2 illustrates the effects of specific mutation altering the native protein function with expected accuracy. SNAP2 gives a score (ranges from −100 strong neutral prediction to +100 strong effect prediction) [10].The results from SNAP2 shows that out of 50 nsSNPs 15 variants (30%) was significant while the rest (70%) show no effect (Table 1). PolyPhen-2 hypothesizes the probable effect of amino acid substitution on structure of protein and function.Variants are classified as probably damaging (probabilistic score0.85 to 1.0), possibly damaging (probabilistic score0.15 to 1.0), and benign (0.0 to 0.15) with specificity and sensitivity values [11]. Among 50 nsSNPs, 22(44%) nsSNPs were probably damaging, 8(16%) as possibly damaging and the remaining (40%) as benign by PolyPhen-2 (Table 1).The prediction accuracy using SIFT, SNAP2 and PolyPhen-2 scores was further validated using PROVEAN. The functional effects of protein sequence variations are illustrated using PROVEAN. The predicted values of amino acid substitutions are above -2.5 are neutral and those below or equal to -2.5 are considered as deleterious [12].Among 50 nsSNPs, 34 amino acid substitutions (68%) were predicted to be neutral (score is above-2.5) and the remaining 16(32%) were having score below or equal -2.5 are assigned to be associated with diseases (Table 1).

SNPs and GO [13], PhD-SNP [14] and PANTHER [15] provide another layer of refinement in nsSNPs characterization. They help predict the impact of mutation on the protein function to conclude disease association. Out of 50 nsSNPs, PhD-SNP and SNPs and GO predicted 17(34%) and 14(28%) nsSNPs respectively to be disease associated and the remaining as neutral. PANTHER predicted 16(32%) nsSNPs as disease-prone and 23(46%) as neutral without data for the remaining nsSNPs (Table 1). The most deleterious nsSNPs was confirmed by concordance. It should be noted that mutants were predicted as deleterious by sequence data aided by SVM models. Thus, a total of 3 variants with F78L (rs200161963), Q644R (rs374267637) and S646F (rs151047872) were found to be deleterious by the above methods.

The selected 3 nsSNPs were analysed by I-mutant 3.0 and NetsurfP to reveal the effect of mutation on protein stability. Thus, the variants were grouped based on free energy change value into positive and negative DDG value groups. Positive DDG value (DDG>0) leads to increased stability whereas negative DDG value (DDG<0) indicates decreased stability with high reliability [16].The variants S646F and Q644R with negative DDG values are considered less stable and F78L with positive DDG value are referred more stable (Table 2).

NetsurfP calculates protein solvent accessibility and assigns secondary structures [17]. The variants S646F, F78L and Q644R were assessed for solvent accessibility and stability. Among these 3 deleterious nsSNPs, 2 variants (S646Fand Q644R) and their respective wild variants were exposed to the surface whereas F78L and its respective wild variant were buried (Table 2). It is known that polar side chains tend to be exposed to the solvent whereas hydrophobic residues tend to be buried in the interior of the protein [19]. The stability of proteins increases with the area of water-accessible hydrophobic surface reduces [20]. It should be noted that the presence of non-polar residues on the surface may reduce stability in the S646F mutant. The leucine side chains cluster together within proteins to stabilize the protein structure through hydrophobic effect. The substitution of phenylalanine by leucine increases protein stability in F78L. The substitution of the polar uncharged residue with a basic charged residue increases the hydrophilicity of surface area in Q644R.

**Figure 1:**
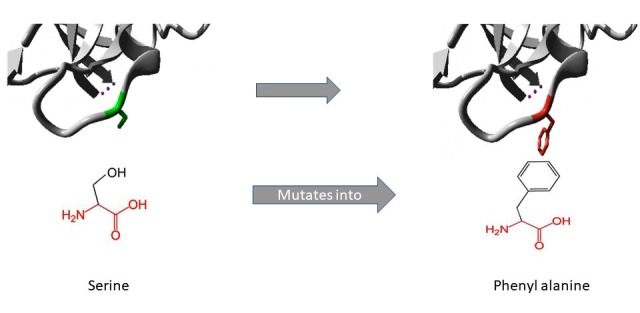
Structural illustration of amino acid substitutions (S646F) in JAK1 protein using the HOPE tool

**Figure 2:**
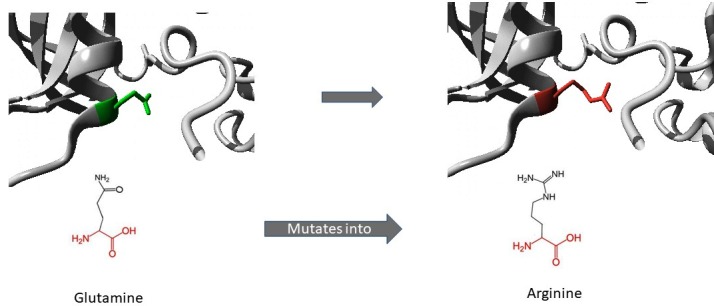
Structural illustration of amino acid substitutions (Q644R) in JAK1 protein shown using the HOPE tool

The 3D structure of protein plays a vital role in unveiling the molecular mechanisms leading to a disease. The evaluation of 3D structure of the mutant protein using HOPE [18] was completed. The mutants S464F (Figure 1) and Q644R (Figure 2) were larger than the wild type and they are situated on the surface of the protein. The F78L mutant was smaller in size and it is located within a domain as annotated in UniProt as FERM (Table 3).The wild-type Q644R is NEUTRAL and the mutant is positively charged. The hydrophobicity of the mutated residue in S464F is more than the wild-type. It is noted in F78L that the mutated residue is located near a highly conserved region. The mutant Q644R shows a substitution of glutamine (polar uncharged) to arginine (basic charged). The mutation of a neutral amino acid with a positively charged residue clashes with the neighboring residues. The large size of the mutated residue on the surface of the protein affects the interaction with other molecules. The combined effect of mutations alters the protein function. Phenylalanine (aromatic) is substituted by leucine (non-polar aliphatic) in F78L. The mutated residue is within FERM and it is crucial for binding with other molecules. The variant with changed properties disrupts the domain with abolishment of function. Further the mutation also results in reduced contact due smaller residue size. The presence of the variant F78L in the highly conserved region is damaging. The substitution of serine (polar uncharged) by phenylalanine (aromatic) in S646F is interesting. This mutation also results in increased size and hydrophobicity of the residue. Residue interacts with different molecules on the surface of the protein. Therefore, its size and location along with changed hydrophilicity has asignificant effect in the structural disruption of the protein leading to altered function.

## Conclusion

JAK1 plays a crucial role in regulating diverse signalling cascades. Increasing evidence has suggested that functional SNPs of JAK1 gene is linked to inflammation and cancer. Several nsSNPs of JAK1 gene are known. However, the functional consequences of the nsSNPs in JAK1 as a result of corresponding structural changes remain unknown. Moreover, discriminating deleterious nsSNPs with potential effects on disease susceptibility from tolerated variants is a major challenge. We report the identification of 3 potentially deleterious nsSNPs (F78L, Q644R and S646F) in the coding region of JAK1 gene. Further studies are needed to validate this preliminary observation 

## Figures and Tables

**Table 1:** Predicted effect of SNPs determined using various tools

SNP	Mutation	SIFT	SNAP2	Polyphen2	Proven	SNPs and GO	PhD-SNP	PANTHER
rs61735631	R506C	Tolerated	Neutral	Possibly Damaging	Neutral	Neutral	Neutral	Disease
rs149968614	V651M	Deleterious	Neutral	Probably Damaging	Neutral	Neutral	Disease	Neutral
rs151047872	S646F	Deleterious	effect	Probably Damaging	Deleterious	Disease	Disease	Disease
rs187043211	N833S	Tolerated	Neutral	Benign	Neutral	Neutral	Neutral	Neutral
rs200049537	R360W	Deleterious	Effect	Probably Damaging	Neutral	Neutral	Disease	Disease
rs202021264	I62V	Tolerated	Neutral	Benign	Neutral	Neutral	Neutral	Neutral
rs34680086	N973K	Tolerated	Neutral	Benign	Neutral	Neutral	Neutral	Neutral
rs115541740	P34A	Tolerated	Neutral	Benign	Neutral	Neutral	Neutral	-
rs117679986	A701V	Tolerated	Effect	Probably Damaging	Deleterious	Neutral	Disease	Neutral
rs137855123	R49Q	Tolerated	Neutral	Benign	Neutral	Neutral	Neutral	-
rs145174573	Y507C	Deleterious	Neutral	Benign	Deleterious	Neutral	Disease	Neutral
rs150021823	R826C	Deleterious	Effect	Possibly Damaging	Deleterious	Neutral	Disease	Disease
rs191513286	L721I	Deleterious	Effect	Probably Damaging	Neutral	Disease	Disease	Disease
rs199886153	R826H	Tolerated	Neutral	Benign	Neutral	Neutral	Neutral	Neutral
rs199914339	N122S	Tolerated	Neutral	Benign	Deleterious	Neutral	Neutral	-
rs200161963	F78L	Deleterious	Effect	Probably Damaging	Deleterious	Disease	Disease	Disease
rs201299733	G592V	Deleterious	Effect	Probably Damaging	Neutral	Disease	Disease	-
rs201432491	S383G	Deleterious	Neutral	Benign	Deleterious	Neutral	Neutral	Neutral
rs201562675	D739E	Deleterious	Effect	Probably Damaging	Neutral	Disease	Disease	Neutral
rs201595595	R506H	Tolerated	Neutral	Benign	Neutral	Neutral	Neutral	Neutral
rs202003827	M101T	Tolerated	Neutral	Benign	Neutral	Neutral	Neutral	-
rs202179869	K860N	Tolerated	Neutral	Benign	Neutral	Neutral	Neutral	Neutral
rs367582687	V985I	Tolerated	Neutral	Benign	Neutral	Neutral	Neutral	Neutral
rs367997081	S71C	Deleterious	Effect	Probably Damaging	Neutral	Disease	Disease	Disease
rs368093469	C1116Y	Tolerated	Neutral	Possibly Damaging	Neutral	Neutral	Disease	Neutral
rs368218410	K1090R	Tolerated	Neutral	Possibly Damaging	Neutral	Neutral	Neutral	-
rs368776025	S512L	Tolerated	Neutral	Possibly Damaging	Deleterious	Neutral	Neutral	Neutral
rs368855745	E903V	Deleterious	Effect	Probably Damaging	Neutral	Disease	Neutral	Disease
rs368904859	D660N	Tolerated	Neutral	Probably Damaging	Deleterious	Neutral	Neutral	Neutral
rs369498502	G741S	Deleterious	Effect	Possibly Damaging	Neutral	Disease	Disease	Disease
rs369863159	K142R	Tolerated	Neutral	Benign	Neutral	Neutral	Neutral	-
rs369910049	H350Q	Tolerated	Neutral	Benign	Deleterious	Neutral	Neutral	Neutral
rs370410494	L225S	Tolerated	Neutral	Probably Damaging	Deleterious	Disease	Disease	Disease
rs370434553	R1113C	Deleterious	Effect	Probably Damaging	Neutral	Disease	Disease	Disease
rs371295663	A862T	Tolerated	Neutral	Benign	Neutral	Neutral	Neutral	Neutral
rs371342094	K580R	Tolerated	Neutral	Probably Damaging	Deleterious	Neutral	Neutral	-
rs372090845	M665I	Deleterious	Neutral	Probably Damaging	Neutral	Neutral	Neutral	Neutral
rs373142876	G307S	Tolerated	Neutral	Benign	Neutral	Neutral	Neutral	Neutral
rs373251481	T178N	Tolerated	Neutral	Benign	Deleterious	Neutral	Neutral	-
rs374267637	Q644R	Deleterious	Effect	Probably Damaging	Deleterious	Disease	Disease	Disease
rs374269002	Y598C	Deleterious	Effect	Probably Damaging	Neutral	Disease	Disease	-
rs374273516	L29V	Tolerated	Neutral	Benign	Neutral	Neutral	Neutral	-
rs374773383	P912A	Tolerated	Neutral	Possibly Damaging	Neutral	Neutral	Neutral	Disease
rs375122732	V433I	Tolerated	Neutral	Probably Damaging	Neutral	Neutral	Neutral	Neutral
rs375353661	V656I	Tolerated	Neutral	Benign	Neutral	Neutral	Neutral	Neutral
rs375956046	V464M	Deleterious	Neutral	Probably Damaging	Deleterious	Neutral	Neutral	Disease
rs375979659	P861T	Tolerated	Neutral	Probably Damaging	Neutral	Neutral	Neutral	Neutral
rs375997338	T263M	Tolerated	Neutral	Possibly Damaging	Deleterious	Neutral	Neutral	Neutral
rs376079085	P1115S	Deleterious	Neutral	Probably Damaging	Neutral	Disease	Neutral	Disease
rs377757935	L710V	Deleterious	Effect	Probably Damaging	Neutral	Disease	Neutral	Disease

**Table 2:** Analysis of selected JAK1 variants using the I-Mutant 2.0 and NetSurfP tools

Mutation	I-Mutant 3.0		NetSurfP		
	DDG	Stability	Class	Relative	Absolute
	value		assignment	surface	surface
				accessibility	accessibility
				(RSA)	(ASA)
S646F	-0.71	Decrease	Exposed	0.638909	74.88009
F78L	0.62	Increase	Buried	0.032513	6.525376
Q644R	-1.39	Decrease	Exposed	0.44884	80.16283

**Table 3:** Amino acid properties assigned using the Hope software

Amino acid	S464F		Q644R		F78L	
properties	Native	Mutant	Native	Mutant	Native	Mutant
Size	Small	Large	Small	Large	Large	Small
Location	Surface	Surface	Surface	Surface	Within	Within
					domain	domain
